# Dynamic Changes in Phenolics and Antioxidant Capacity during Pecan (*Carya illinoinensis*) Kernel Ripening and Its Phenolics Profiles

**DOI:** 10.3390/molecules23020435

**Published:** 2018-02-16

**Authors:** Xiaodong Jia, Huiting Luo, Mengyang Xu, Min Zhai, Zhongren Guo, Yushan Qiao, Liangju Wang

**Affiliations:** 1 College of Horticulture, Nanjing Agricultural University, Nanjing 210095, Jiangsu, China; 2012204006@njau.edu.cn; 2 Institute of Botany, Jiangsu Province and Chinese Academy of Sciences, Nanjing 210014, Jiangsu, China; luohuiting@cnbg.net (H.L.); xumengyang@cnbg.net (M.X.); zhaimin@cnbg.net (M.Z.); zhongrenguo@aliyun.com (Z.G.)

**Keywords:** *Carya illinoinensis* (Wangenh.) K. Koch, phenolics, antioxidant capacity

## Abstract

Pecan (*Carya illinoinensis*) kernels have a high phenolics content and a high antioxidant capacity compared to other nuts—traits that have attracted great interest of late. Changes in the total phenolic content (TPC), condensed tannins (CT), total flavonoid content (TFC), five individual phenolics, and antioxidant capacity of five pecan cultivars were investigated during the process of kernel ripening. Ultra-performance liquid chromatography coupled with quadruple time-of-flight mass (UPLC-Q/TOF-MS) was also used to analyze the phenolics profiles in mixed pecan kernels. TPC, CT, TFC, individual phenolics, and antioxidant capacity were changed in similar patterns, with values highest at the water or milk stages, lowest at milk or dough stages, and slightly varied at kernel stages. Forty phenolics were tentatively identified in pecan kernels, of which two were first reported in the genus *Carya*, six were first reported in *Carya illinoinensis*, and one was first reported in its kernel. The findings on these new phenolic compounds provide proof of the high antioxidant capacity of pecan kernels.

## 1. Introduction

The pecan [*Carya illinoinensis* (Wangenh.) K. Koch], originally from North America, is one of the most important nut crops worldwide. It was introduced into China over 100 years ago and has attracted great attention this decade as a nut full of nutritional value [1,2]. Recently, more and more attention has been given to the high antioxidant capacity of pecan kernels. Wu et al. [3] screened over 100 commonly consumed foods in the USA and found that pecan kernels have the highest TPC and antioxidant capacity among the nut group, higher than many other fruits and vegetables rich in phenolics. Another study on proanthocyanidins (which also belong to the phenolics family) content in 98 common foods showed that pecans had the second highest content [4]. These results highlight the possibility of considering pecans as a nutritional food that has high phenolic content and good antioxidant capacity.

The strong antioxidant capacity of pecan kernel mainly comes from the phenolic compounds [5]. Phenolics found in pecan kernels were mainly flavan-3-ols [6], anthocyanidins [7], proanthocyanidins [4], phenolic acid [8], and their sugar-containing glycosides [9] or polymeric tannins with degrees up to 10 [4]. Phenolics were reported to have strong antioxidant activities, which can capture radical particles to reduce the risks of chronic diseases, including cardiovascular [10], cancer [11], and diabetes [12], and protect other components from oxidation [5,13,14]. Phenolics have also been proven to have antiviral [15] and antihypertensive activities [13].

It is nutritionally important to know the composition of phenolics and how they accumulate during nut maturation. The dynamic changes of soluble sugar, protein, fiber, moisture, mineral elements [16], fatty acid, tocopherol, xanthophyll [17], phytosterol, and phytostanols [18] in pecan kernels during ripening have been studied before. However, profiles of important constituents—phenolics—and their antioxidant capacity in pecans during ripening are still lacking. Previous research on pecan kernels using a column separation coupled LC-ESI-MS method led to the isolation of 35 phenolic compounds [9]. UPLC-Q/TOF-MS were more sensitive and gave more structural information, which will ease the identification of complex phenolics. Phenolics and their antioxidant capacity were compared among several pecan cultivars of mature kernels grown in the USA [6,19], but no study of their changes during the whole kernel development has been conducted before. 

Therefore, five cultivars that are now cultivated over large areas in China were chosen to analyze the changes in phenolics and antioxidant capacity from the appearance of the kernel to full maturity; phenolics were also identified using UPLC-Q/TOF-MS. Of the five cultivars, three (Pawnee, Stuart, and Wichita) were introduced from the USA, while the other two (Jinhua and Shaoxing) were selected from local superior seedlings. Cultivar Jinhua was selected in 1980 from a seedling at a kindergarten where there used to be a hospital opened by an American doctor, at Jinhua City, Zhejiang Province located in southeast China [1]. Cultivar Shaoxing was selected in 1980 from another superior seedling tree located in Shaoxing City, Zhejiang Province [1]. The purposes of this study were to investigate the dynamic changes of phenolics and antioxidant capacity in pecan kernels during kernel development and analyze the phenolics profiles of pecan kernels. 

## 2. Results and Discussion

### 2.1. Changes of Total Phenolic Content, Condensed Tannins, and Total Flavonoid Content

**Figure 1:**
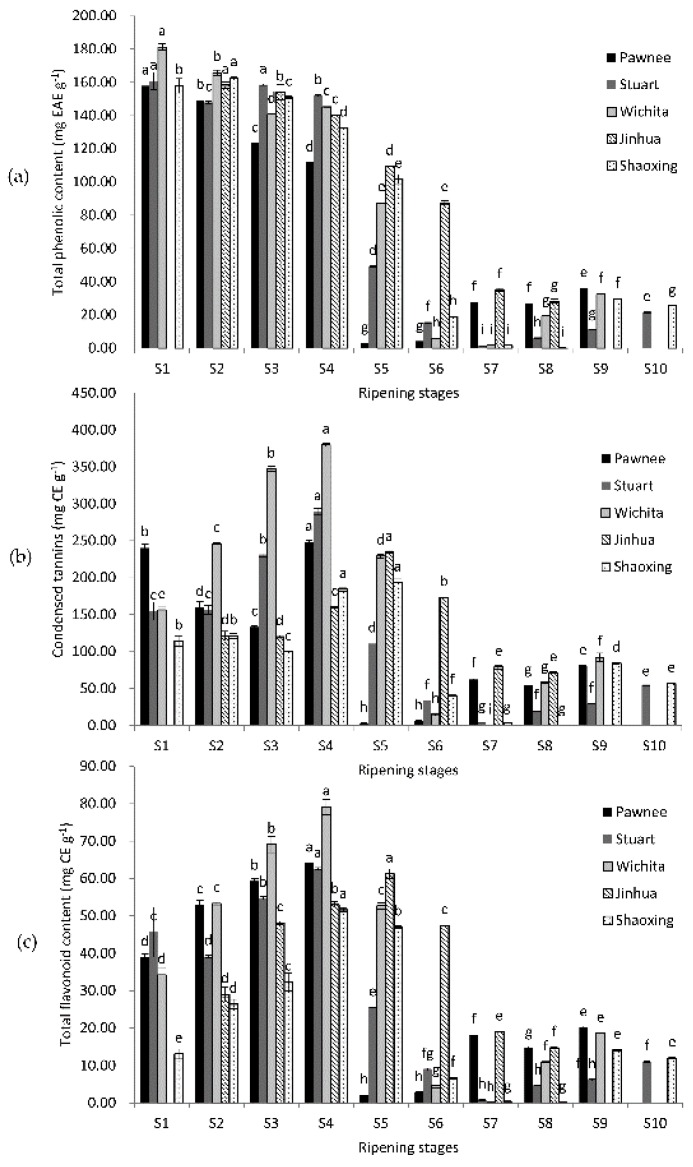
Changes in total phenolic content (**a**); condensed tannins (**b**); and total flavonoid content (**c**) during the ripening of pecan kernels. Each value is a mean ± standard deviation of triplicate analysis results of different samples. Means with different letters in each line were significantly different according to the multiple-range Tukey’s test at *p* < 0.05.

The highest TPC appeared at the water stages (S1–S3) of fruit ripening of all five cultivars (Figure 1). Then, TPC declined quickly and reached the lowest point at the mike stage (S4–S5) or dough stages (S7–S8), with a slightly bounce at the kernel stages (except Jinhua). CT and TFC shared similar change patterns: the highest value appeared at milk stages (S4–S5) and the lowest value showed at dough stages (S6–S8). The highest TPC value was in Wichita (181.28 mg EAE g^−1^) at S1, while the lowest was in Shaoxing at S8 (0.23 mg EAE g^−1^). Both the maximum CT and TFC values appeared at S4 of Wichita (379.85 and 79.17 mg CE g^−1^), while the minimum values showed at S7 of Wichita (0.3 and 0.1 mg CE g^−1^). The TPC, CT, and TFC of mature kernels were 11.36–29.50 mg EAE g^−1^, 29.5–84.3, and 6.1–14.8 mg CE g^−1^, respectively. TPC, CT, and TFC increased in over-ripe samples, except for Jinhua, which did not have over-ripe samples, and Shaoxing, in which they all decreased. The TPC, CT, and TFC values were all significantly affected by cultivars.

Previous reports mainly focused on mature pecan kernels. Ellagic acid was chosen as the standard for TPC in this experiment because it is more representative of the type of phenolic compounds that exist in pecan kernels [19]. Results of TPC of mature pecan kernels in previous reports were mostly around 18.2–26.2 mg EAE g^−1^ (using ellagic acid as standard) [19] or 10–20 mg GAE g^−1^ (using gallic acid as a standard) [3,8,20,21,22,23,24]. Though different standards made it hard to compare, we still see that our TPC values of mature kernels were in a similar range to most reports. Slight differences may be caused by the variation in geographic location, climate difference, or cultivation techniques. When comparing our CT values of mature kernels with previous reports, the result of cultivar Stuart was lower than previous reports, while results of cultivars Pawnee, Wichita, Jinhua, and Shaoxing were similar to previous reports [8,20,22,24,25,26]. When comparing TFC values, our results were more similar to the reports of de la Rosa et al. [20] and Yang et al. [22]. Changes in TPC and TFC were widely investigated in fruits such as grapes and strawberries [27,28], which usually decreased along with ripening, but rarely in nuts. The values of CT were higher than TPC values in pecan kernels, which is consistent with previous research on mature pecans [6,8,20]. These results may attribute to the overestimation caused by the higher extinction coefficient of polymeric tannins to free catechin [29]. These results also suggested that pecan kernels contain highly polymeric tannins. 

### 2.2. Antioxidant Capacity

**Figure 2:**
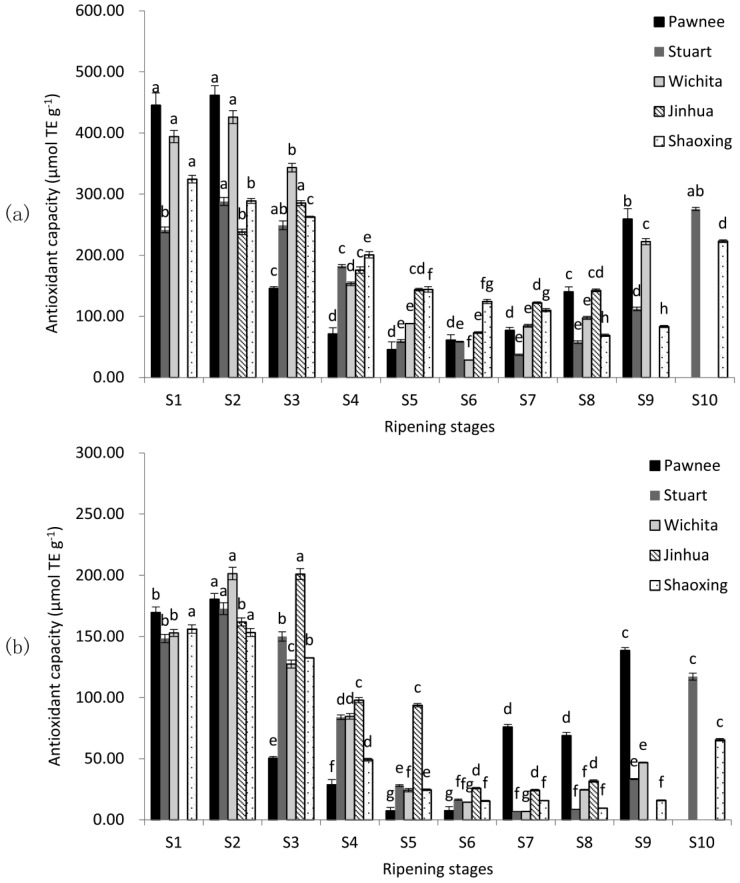
Changes in DPPH (**a**) and ABTS (**b**) during the ripening of pecan kernels. Each value is a mean ± standard deviation of triplicate analysis results of different samples. Means with different letters in each line were significantly different according to the multiple-range Tukey’s test at *p* < 0.05.

Antioxidant capacity was valued using two in vitro assays, including the 2, 2-diphenyl-1-picrylhydrazyl-based assay (DPPH) and the 2, 2′-azino-bis (3-ethylbenzo-thiazoline-6-sulphonic acid) diammonium salt-based assay (ABTS) (Figure 2). The highest antioxidant capacity levels were observed at the water stages in all five cultivars of both assays, and declined significantly (*p* < 0.05). When mature (Pawnee, Wichita, and Jinhua at S8; Stuart and Shaoxing at S9), antioxidant capacities of pecan cultivars were in the descending order Pawnee ≈ Jinhua > Stuart > Wichita > Shaoxing in DPPH assay and Pawnee > Jinhua ≈ Stuart > Wichita > Shaoxing in ABTS assay. The antioxidant capacity was affected significantly by cultivars. Among the five cultivars, Pawnee had the highest DPPH of 461.80 μmol TE g^−1^ (S2) and Wichita had the highest ABTS of 201.49 μmol TE g^−1^ (S2), while Stuart had the lowest DPPH of 37.24 μmol TE g^−1^ and ABTS of 6.74 μmol TE g^−1^ (S7). Over-ripeness increased the antioxidant capacity in all cultivars (except Jinhua).

Although the high antioxidant capacity of pecan kernel was reported before [3,4,10], no report of their changes was available during kernel ripening. As shown in Figure 2, the antioxidant capacity followed a similar trend to TPC, CT, and TFC during pecan nut ripening and the antioxidant capacity of immature samples was higher than that of mature samples. Since dynamic changes of pecan kernel antioxidant capacity had not been investigated before, we can only compare our results of mature kernels with previous reports on pecans. Our results for mature kernels were similar to those reported by Villarreal-Lozoya et al. [6] and de la Rosa et al. [8].

### 2.3. Identification of Phenolics in Pecan Kernels Using UPLC-Q/TOF-MS

UPLC-Q/TOF-MS was used to identify the phenolics in pecan kernels. Cultivar Pawnee was chosen due to its relatively high antioxidant capacity among the five cultivars. Pecan kernels of Pawnee of 10 developmental stages were pooled and analyzed using UPLC-Q/TOF-MS. Both the positive and the negative mode were used to identify phenolic compounds, while results showed that the negative mode was more suitable for phenolics. So, subsequent analyses were carried out using negative mode.

A total of 40 phenolics were tentatively identified in pecan kernels, of which two were first reported in the genus *Carya*, six were first reported in species *Carya illinoinensis* and one was first reported in pecan kernels. The retention time (RT), molecular ion, fragment ions, molecular formula, and mass measurement errors (∆m) are shown in Table 1.

More than half of the phenolics identified in pecan kernel were ellagic acid derivatives. The free ellagic acid (peak 20) was detected at RT of 6.36 min with a [M − H]^−^ of *m*/*z* 300.9961 and confirmed by comparing with commercial standard. Ellagic acid hexoside (Peak 19) and ellagic acid pentose (peak 21) can easily be found by the characteristic fragment ion at *m*/*z* 301 along with the neutral losses of 162 amu (the loss of hexoside) and 132 amu (the loss of pentose), respectively. Peak 29 was tentatively identified as ellagic acid rhamnoside because of the same fragment ion at *m*/*z* 301 and a neutral loss of 146 amu [36]. Peak 25 was assigned as ellagic acid rutinoside for a neutral loss of 308 amu, which corresponded to the loss of a rhamnose-glucose structure. To the best of our knowledge, both ellagic acid rhamnoside and ellagic acid rutinoside were identified in the *Carya* genus for the first time.

Ellagic acid is formed from the condensation reaction of two gallic acids. Galloyl-linked ellagic acid derivatives can also be found in pecan kernels. Peak 33 with [M − H]^−^ ion at *m*/*z* 585 produced ions at *m*/*z* 433 (M − H − 152, loss of galloyl) and *m*/*z* 301 (M − H − 284, loss of galloyl and pentose) was assigned as ellagic acid galloyl pentose. Peak 23 with [M − H]^−^ ion at *m*/*z* 615 produced ions at *m*/*z* 463 (M − H − 152, loss of galloyl) and *m*/*z* 301, which corresponded to the deprotonated ellagic acid, was assigned as digalloyl ellagic acid.

Similar methyl ellagic acid derivatives can be identified with the characteristic fragment ion at *m*/*z* 315, including methyl ellagic acid (peak 30), methyl ellagic acid hexoside (peak 24), methyl ellagic acid pentose (peak 31), and methyl ellagic acid galloyl pentose (peak 36, 37 and 38). Dimethyl ellagic acid derivatives added an extra 15 amu (another methyl group) to form the main ion at *m*/*z* 328. Several peaks that contained this kind of structure were assigned, including dimethyl ellagic acid (peak 39 and 40) and dimethyl ellagic acid hexoside (peak 27).

**Figure 3:**
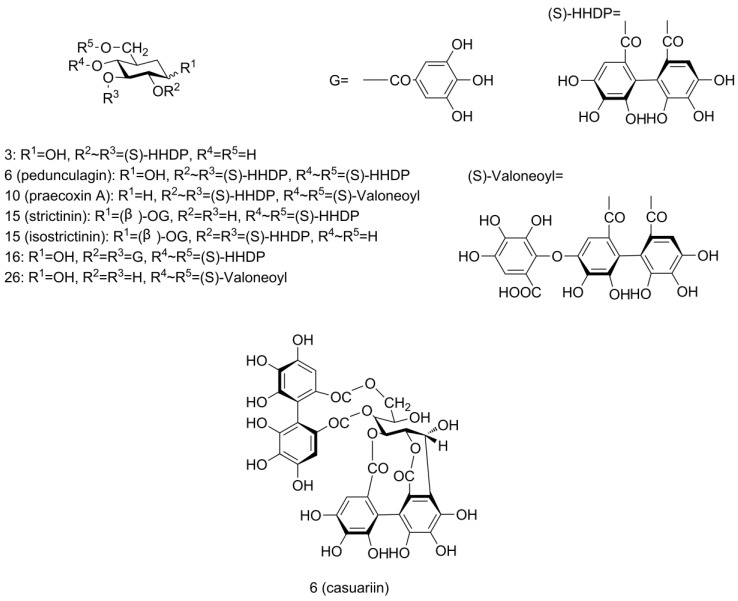
Structures of ellagitannins tentatively identified in pecan kernels, including peaks 3, 6, 10, 15, 16, and 26.

Another ellagic-acid-related derivative family was the ellagitannins (ETs), which contain the hexahydroxydiphenoyl (HHDP) group (Figure 3). HHDP-glucose was found at RT 0.94 min with [M − H]^−^ ion at *m*/*z* 481 (peak 3), fragment ion at *m*/*z* 301 and a neutral loss of 180 amu. 

Bis-HHDP-glucose (pedunculagin/casuariin isomer), which contains two HHDP groups, was found with [M − H]^−^ ions at *m*/*z* 783 (peak 6 and 9). MS/MS spectra yielded fragment ions at *m*/*z* 481 (M − H − 302, loss of HHDP) and 301 (M − H − 482, loss of HHDP-glucose), confirmed the assignment. Peak 10 was found to yield similar ions at *m*/*z* 783, 481, 301 as bis-HHDP-glucose (peak 6 and 9) along with extra ions at *m*/*z* 907 (M − H − 44, loss of carboxyl) and 951 [M − H]^−^. From this fragmentation pattern, we can see that another gallic acid group was linked in this compound. So, this ET was assigned the name HHDP-valoneoyl-glucose (praecoxin A/platycariin isomer). A peak showing [M − H]^−^ signal at *m*/*z* 649 (peak 28) with fragment ions at *m*/*z* 605 (M − H − 44, loss of carboxyl), 481 (M − H − 168, loss of gallic acid) and 301 (M − H − 348, loss of gallic acid and glucose) was assigned as valoneoyl-glucose. To our knowledge, bis-HHDP-glucose (pedunculagin/casuariin isomer), HHDP-valoneoyl-glucose (praecoxin A/platycariin isomer), and valoneoyl-glucose were identified for the first time in pecans. They had been reported previously in walnut kernels [30,33].

Galloyl-HHDP-glucose (strictinin/isostrictinin) was identified at RT 4.59 min with [M − H]^−^ ion at *m*/*z* 633 (peak 16) and fragment ion at *m*/*z* 301 (M − H − 332, loss of galloyl-glucose). Another peak with [M − H]^−^ ion at *m*/*z* 785 (peak 17) was detected. Fragment ions were at *m*/*z* 483 (M − H − 302, loss of HHDP) and 301 (M − H − 484, loss of digalloyl group and glucose), which corresponded to deprotonated digalloyl glucose and deprotonated HHDP, respectively. This fragmentation pattern complied well with digalloyl-HHDP-glucose (tellimagrandin I).

Another dominant class of compounds in pecan kernels was catechin and its derivatives. (+)-Catechin (*m*/*z* 289, peak 15), (−)-epicatechin (*m*/*z* 289, peak 18), (epi)catechin gallate (*m*/*z* 441, peak 26), epigallocatechin gallate (*m*/*z* 457, peak 34), and catechin hexoside (*m*/*z* 451, peaks 7 and 8) were identified by comparing MS/MS fragment ions with reference reports or commercial standards. Procyanidin dimer B-type [(epi)catechin→B→(epi)catechin] was identified with [M − H]^−^ ions at *m*/*z* 577 (peak 11) and fragment ions at *m*/*z* 425 (M − H − 152, loss of galloyl), which complied with the reports in pecan and peanut skins [9,37]. B-type means that two (epi)catechin structures are linked together through C4→C8 or C4→C6, in which C4→C8 is more common due to low space resistant. Peak 13 yielding [M − H]^−^ ion at *m*/*z* 865 and fragment ions at *m*/*z* 577 (M − H − 288, loss of catechin) and 289 (M − H − 576, loss of procyanidin dimer) was assigned the name procyanidin trimer (C1) [(epi)catechin→B→(epi)catechin→B→(epi)catechin] [9,37].

Gallic acid derivatives can also be found in pecan kernels. The free gallic acid (peak 4) can be identified at RT of 1.15 min and confirmed by comparing with a commercial standard. Several other gallic acid derivatives were also identified, including gallic acid hexoside (*m*/*z* 331, peak 2), methyl gallate (*m*/*z* 183, peak 12), and digalloyl-glucose (*m*/*z* 483, peak 14). Methyl gallate was previously found in pecan leaves [31], while this is the first time it has been reported in pecan kernels.

Two dicarboxylic acid derivatives were detected in pecans for the first time. Glansreginin A and B were identified by the characteristic [M − H]^−^ ions at *m*/*z* 592 (peak 32) and 565 (peak 22) and fragment ions at *m*/*z* 403/343/241, which were identical to previous reports [33]. These two compounds were first isolated and elucidated in walnut kernels [38]; they have also been found in hazelnut kernels [39].

### 2.4. Quantification of Phenolics in Pecan Kernels Using HPLC

Although UPLC-Q/TOF-MS is very sensitive and suitable for identification of compounds, due to its relatively low peak resolution within a short procedure, HPLC was used to quantify the dynamic changes of several representative phenolics. 

**Figure 4:**
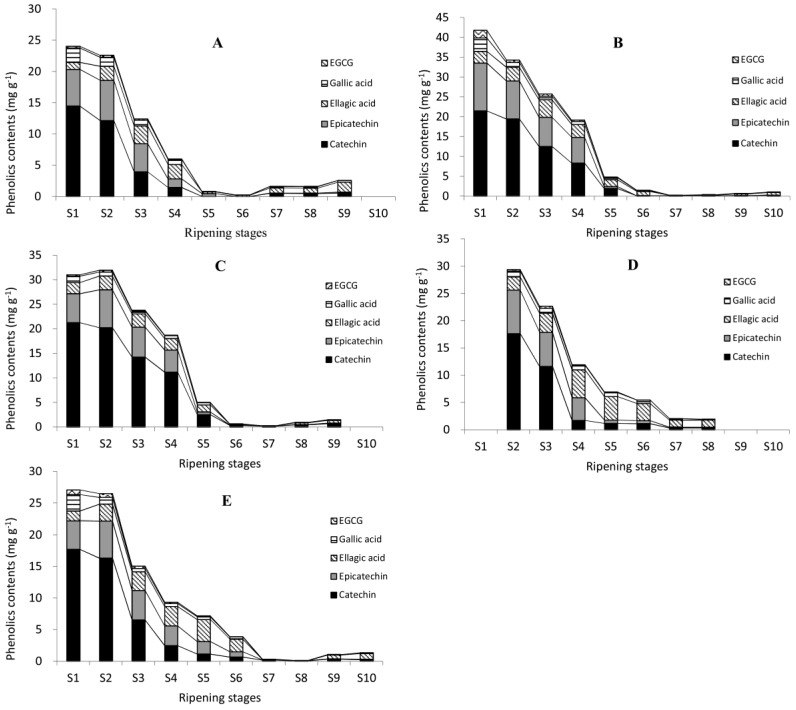
Changes in five representative individual phenolic contents during the ripening of pecan kernels. Pawnee (**A**); Stuart (**B**); Wichita (**C**); Jinhua (**D**); and Shaoxing (**E**). Black lines on the top of different columns of different images are trend lines of different phenolics.

The contents of gallic acid, (+)-catechin, (−)-epicatechin, EGCG, and ellagic acid were analyzed in five cultivars of 10 developmental stages (Figure 4). The contents of five individual phenolics were all highest at water stages, lowest at dough stages, and varied at the kernel stages, similar to the change patterns of TPC, CT, and TFC. Similar results can be found in research on *Anacardium occidentale* L., while phenolic contents (like gallic acid and EGCG) decreased significantly from unripe to medium-ripe and ripe [40]. This fall may be attributable to their conversion to other downstream forms such as esterified or glycoside forms, along with the nut development. It may also attribute to the high proportion of testa at immature stages. In immature pecan kernels, the proportion of testa was high, and decreased gradually with the nut development. Senter et al. reported firstly that pecan testa might contain powerful antioxidant phenolics and act as a natural screen to prevent infection by viruses, bacteria, and molds [41]. They speculated that phenolics existed mainly in the outer testa or pellicle of the pecan kernels [42]. Research showed that contents of (+)-catechin and (−)-epicatechin in the testa of cashew nut were 20 times and five times higher than in green tea and dark chocolate, respectively [43,44]. Further experiments are needed to obtain more accurate data on phenolics in different organs of pecan kernels.

In immature pecan kernels, the most abundant phenolic was (+)-catechin, followed by (−)-epicatechin, while EGCG had the lowest content. The content of (+)-catechin was much higher, e.g., over 10 times (17.7100 mg g^−1^ at S1) higher than ellagic acid (1.4982 mg g^−1^ at S1) in Shaoxing, than other phenolics during immature stages. This was also probably due to the fact that they were used as building blocks to form proanthocyanidins. Two or three catechin units linked together formed proanthocyanidins (dimers and trimers), while three or more catechin units linked together formed oligomeric proanthocyanidins [45]. Both proanthocyanidins and oligomeric proanthocyanidins are abundant in pecan kernels. Two proanthocyanidins were identified in this experiment. So, catechin was very likely to be stored at a high concentration at the beginning of kernel formation for future use.

In mature kernels (Pawnee, Wichita, and Jinhua at S8; Stuart and Shaoxing at S9), the contents of ellagic acid were highest in Pawnee, Stuart, Jinhua, and Shaoxing, but in Wichita the content of (+)-catechin was the highest. This result was consistent with previous reports on pecans, in which most of them reported the most abundant free phenolic was ellagic acid [9,19], while some reported it to be (+)-catechin [46]. Phenolics are secondary metabolites, so environmental factors such as temperature and sun irradiation could affect their concentration [47]. For example, a walnut husk cultivated at higher altitude contained higher total phenolic content than one cultivated at a lower altitude [48]. The temperature and sunshine duration during pecan kernel mature were recorded; however, the effects of these environmental factors on phenolic contents await further study.

### 2.5. The Correlation of Antioxidant Capacity and Phenolics

A correlation was determined between antioxidant capacity and individual phenolics (Table 2). TPC, CT, TFC, and individual phenolics were all significantly correlated with antioxidant capacity, especially of (+)-catechin, (−)-epicatechin, and TPC. (+)-Catechin had the highest correlation coefficient with antioxidant capacity, followed by (−)-epicatechin among individual phenolics. Coefficients between TPC and antioxidant capacity were higher than those of CT and TFC. This result was consistent with previous reports on mature pecans [8,19,49,50]. (+)-Catechin and (−)-epicatechin might be the main components responsible for the high antioxidant capacity of pecan kernels. It is reported that phenolics have high scavenging abilities with DPPH free radicals [51]. The value of DPPH in our experiment was higher than that of ABTS (Figure 2); similar results can be found in previous pecan reports [8,50]. ABTS was measured at the near-infrared area of 734 nm, which cut off a lot of interference from other absorbing components such as sugars [51]. This might lead to a more precise result and might be the reason for its higher correlation coefficient with phenolics compared to DPPH (Table 2).

Phenolics identified in pecan kernels mainly included derivatives of ellagic acid, gallic acid, and catechin. Seven ETs and two proanthocyanidins were identified in this experiment. Both ETs and proanthocyanidins were reported to have a high antioxidant capacity [52]. However, due to the lack of quantification of other phenolics identified by UPLC-Q/TOF-MS, the correlations of antioxidant capacity with these phenolics were not analyzed. Further experiments will focus on that aspect to elucidate the relations between the complex phenolics and antioxidant capacity.

## 3. Materials and Methods

### 3.1. Pecan Samples

The experimental trees were planted in the scientific orchards of the Institute of Botany, Jiangsu Province and Chinese Academy of Sciences, under normal management of water and fertilizer. Nuts of five pecan cultivars, including Pawnee, Stuart, Wichita (introduced cultivars), Jinhua, and Shaoxing (local seedling selection cultivars), were sampled at 10 developmental stages from 85 to 175 days after full blossoming at 10-day intervals in 2016 (Table 3). The daily mean temperatures of sampling interval were 18.27–32.09 °C and the daily mean sunshine durations of sampling interval were 1.00–10.35 h. The total sampling times of five cultivars varied due to the differences in fruit development. Cultivars Pawnee, Wichita, and Jinhua were fully mature at S8, while cultivars Stuart and Shaoxing were at S9. Fruits were sampled from as soon as the kernel appeared until it was mature. Additionally, fruits were allowed to stay on the tree for 10 days after maturity to evaluate the effect of over-ripeness. Nine healthy pecan trees (10 years old) of each cultivar were selected as sample trees. Two healthy nuts were hand-picked from the four directions, east, south, west, and north, which gave a total of eight nuts from each tree. Nuts from every three trees were combined together to form three biological replicates, which contained 24 nuts in each replicate and 72 nuts for each stage of a single cultivar. The samples were immediately placed in an ice box and transported back to the laboratory. The kernels were separated manually and stored at −70 °C for analysis. Before use, samples were powdered and homogenized.

### 3.2. Chemicals

Solvents including hexane, acetone, and acetonitrile were HPLC grade and purchased from Merck (Merck Chemicals, Darmstadt, Germany). Sodium carbonate, Folin-Ciocalteu regent, vanillin regent, sodium nitrite, aluminum chloride, NaOH, glacial acetic acid, 2,2-diphenyl-1-picrylhydrazyl (DPPH), 2,2′-azino-bis(3-ethylbenzo-thiazoline-6-sulphonic acid) diammonium salt (ABTS), 6-hydroxy-2,5,7,8-tetramethylchroman-2-carboxylic acid (trolox), gallic acid, ellagic acid, (+)-catechin, (−)-epicatechin, rutin, and EGCG were purchased from Sigma-Aldrich (St. Louis, MO, USA).

### 3.3. Sample Extraction

Pecan kernels were dried and then defatted with hexane. Phenolic compounds were extracted according to the methods of de la Rosa et al. [8] and Villarreal-Lozoya et al. [6], with slight modifications. After volatilization of the solvent residue, defatted pecan kernels (1 g) were placed in a 50-mL centrifuge tube with a screw cap; 20 mL of 80% acetone was added, and the mixture was rested overnight, ultrasonic extracted for 2 h, and centrifuged at 6000 rpm for 10 min at 4 °C (Hettich, Andreas Hettich GmbH & Co. KG, Tuttlingen, Germany). The kernel residues were ultrasonically extracted again with another 20 mL of 80% acetone, and the supernatants were combined. Solvents were removed by nitrogen blowing (Anpel nitrogen evaporator, Anpel Laboratory Technologies Inc., Shanghai, China) at 50 °C and lyophilized (Songyuan Huaxing LGJ-12, Beijing Songyuan Huaxing Technology Develop Co., Ltd, Beijing, China). Dried extracts were dissolved in methanol and stored at −20 °C until use.

### 3.4. Total Phenolic Content

TPCs were determined according to the method of de la Rosa et al. [8] and Robbins et al. [19]. Methanol extract (10 μL) was transferred to a 10-mL centrifuge tube and 2 mL of 7.5% (*w*/*v*) sodium carbonate and 2.5 mL of 10% (*v*/*v*) Folin–Ciocalteu regent were added and the mixture reacted in a water bath at 50 °C for 15 min in the dark. After cooling to room temperature, the absorbance was measured at 760 nm using UV spectrophotometer (Shimadzu UV-2100, Shimadzu Corporation, Kyoto, Japan). Ellagic acid was used as a standard reference, and the results were expressed as milligrams of ellagic acid equivalent (EAE) per gram of defatted kernel weight (mg EAE g^−1^). All blanks used in this article were made using pure methanol without sample extracts, and treated along with samples using the same method at the same time. All analyses in this article were performed in triplicate with three biological replicates.

### 3.5. Condensed Tannins

CT assays were performed using a vanillin assay [8]. The methanol extract (200 μL) was transferred to a 10-mL centrifuge tube, and then 2.5 mL vanillin regent (5 g of reagent and 1 L of 4% HCl methanol, *v*/*v*) was added and reacted at room temperature for 20 min in the dark. Absorbance was measured at 500 nm using a UV spectrophotometer. (+)-Catechin was used as a standard reference, and the results were expressed as milligrams of (+)-catechin equivalents (CE) per gram of defatted kernel weight (mg CE g^−1^).

### 3.6. Total Flavonoid Content

TFCs were measured according to the method of de la Rosa et al. [20] with some modifications. Briefly, methanol extract (100 μL) was transferred to a 10-mL centrifuge tube, 1 mL of 5% sodium nitrite (*w*/*v*) was added, and the mixture was incubated for 5 min in the dark. Then, 1 mL of 10% aluminum chloride (*w*/*v*) was added and incubated for 3 min. After adding 5 mL of 4% NaOH (*w*/*v*) and incubating in the dark for 30 min, absorbance was measured at 510 nm using UV spectrophotometer. (+)-Catechin was used as a standard reference, and the results were expressed as milligrams of (+)-catechin equivalents per gram of defatted kernel weight (mg CE g^−1^).

### 3.7. Antioxidant Capacity

Firstly, the antioxidant capacity of pecan kernels was measured using a DPPH assay according to a previous report [53], and modified as described by Prado et al. [50] and Villarreal-Lozoya et al. [6]. A DPPH radical solution was prepared by dissolving 39.43 mg DPPH in 1 L methanol, and storing at 4 °C before use. Methanol extract (10 μL) was transferred into 15-mL centrifuge tube and 4 mL of DPPH radical solution was added. The centrifuge tubes were kept in the dark for 30 min for a scavenging reaction. Then, absorbance was measured at 515 nm with a UV spectrophotometer. Methanol without sample was used as a blank and treated with the same method as the samples. Absorbance of blank was subtracted from each sample. Trolox was used as a standard reference, and the results were expressed as μmol trolox equivalents (TE) per gram of defatted kernel weight (μmol TE g^−1^).

The ABTS assay was carried out according to previous reports [21,50]. Briefly, an ABTS˙^+^ solution (7.0 mM) was prepared by dissolving 38.36 mg of ABTS in 10 mL deionized water, mixed with a potassium persulfate solution (2.45 mM) at a ratio of 1:1 (*v*/*v*); it was kept in the dark for at least 16 h to form radicals. Before use, the ABTS˙^+^ solution was diluted with ethanol to an absorbance of 0.70 ± 0.05 at 734 nm with UV spectrophotometer. Then, 40-μL extracts were mixed with 2 mL ABTS˙^+^ solution, and the absorbance was measured at 734 nm after 6 min. Methanol without sample was used as a blank and treated with the same method as the samples. Absorbance of the blank was subtracted from each sample. Trolox was used as a standard reference, and the results were expressed as μmol trolox equivalents per gram of defatted kernel weight (μmol TE g^−1^).

### 3.8. UPLC-Q/TOF-MS

Methanol extracts of pecan kernels (Pawnee) at 10 developmental stages were mixed together. Mixed samples were analyzed using a Waters ACQUITY UPLC system (Waters, Milford, MA, USA) coupled with a Waters ACQUITY Q-TOF mass spectrometer (Waters, Milford, MA). An ACQUITY UPLC BEH C_18_ column (2.1 mm × 50 mm, 1.7 um, Waters, Milford, MA, USA) was used at 35 °C. The mobile phase was composed of acetonitrile (A) and water containing 0.1% formic acid (B, *v*/*v*) at a flow rate of 0.4 mL min^−1^. The solvent gradient was as follows: 5–10% A at 0–4 min, 10–50% A at 4–13 min, and 50–100% A at 13–20 min. Both negative and positive ion modes were used in this experiment, with a full mass scan at *m*/*z* 100–1500. The parameters of MS were set as follows: capillary voltage, 3 kV; source temperature, 120 °C; desolvation temperature, 350 °C; cone voltage, 50 V; cone gas flow, 50 L h^−1^; desolvation gas flow, 600 L h^−1^. Rutin was used as the lock mass.

The identification of compounds were carried out first by comparing the retention time, [M − H]^−^ ion, MS/MS fragments, and UV/Vis spectra data with previous reports on pecans to find known compounds. Then, available commercial standards including gallic acid, (+)-catechin, (−)-epicatechin, EGCG, and ellagic acid were used to further confirm these compounds. Compounds not found in the reports on pecans were searched for in the reports on the genus *Carya*, and then the family Juglandaceae, together with the Kyoto Encyclopedia of Genes and Genomes (KEGG) database. Peaks found in all three biological replicates with mass measurement errors (∆m) lower than 10 ppm were recorded in Table 1. 

### 3.9. HPLC

HPLC were performed on Agilent 1100 HPLC (Agilent Technologies, San Diego, CA, USA) with a C_18_ column (Gemini, 250 mm × 4.6 mm, 5 μm particle size, Phenomenex, Torrance, CA, USA) conducted at 35 °C. The mobile phase was composed of acetonitrile (A) and water containing 2% of glacial acetic acid (B, *v*/*v*) a flow rate of 1 mL min^−1^. The solvent gradient was as follows: 5–37% A at 0–40 min. Detection wavelengths were 250 nm (ellagic acid) and 280 nm (other four phenolics). Standard compounds were analyzed under the same conditions and standard curves were drawn; then contents of phenolic compounds were calculated using regression equations.

### 3.10. Statistical Analysis

The data were analyzed using Excel and SPSS (version 18.0) software. MS data was processed using Mass Lynx (version 4.1, Waters MS Technologies, Manchester, UK) software. All analyses were performed in triplicate. One-way analysis of variation (ANOVA) was performed and the mean values were compared by Turkey’s test; differences at *p* < 0.05 were considered to be significant. Correlations were also obtained using SPSS software. 

## 4. Conclusions

Dynamic changes in TPC, CT, TFC, phenolics, and antioxidant capacity of five widely cultivated Chinese-grown pecan cultivars (Pawnee, Stuart, Wichita, Jinhua, and Shaoxing) were analyzed. Similar dynamic change patterns were found: values were highest at the water or milk stages, lowest at milk or dough stages, and slightly varied at kernel stages. Phenolics profiles were also performed using pecan kernels. Using UPLC-Q/TOF-MS, 40 phenolics were tentatively identified in pecan mixed kernels, of which ellagic acid rhamnoside and ellagic acid rutinoside were first reported in the genus *Carya*; six phenolics, i.e., bis-HHDP-glucose (pedunculagin/casuariin isomer, two isomers), HHDP-valoneoyl-glucose (praecoxin A/platycariin isomer), valoneoyl–glucose, glansreginin A, and glansreginin B were first reported in pecans; and methyl gallate was first reported in pecan kernels. (+)-Catechin was found to be the most abundant phenolic in immature kernels and may convert to proanthocyanidins and oligomeric proanthocyanidins, which may be the major antioxidant constituents at the kernel stages. This experiment only carried on for one ripening cycle; further research is needed using samples of more cycles to characterize more precisely the tendency for phenolic compositions. Further research is also needed to investigate the accumulation of these newly found phenolics in pecan kernels and testa, and their relationship with the antioxidant capacity of pecan kernels.

## Figures and Tables

**Table 1:** Phenolics tentatively identified in mixed pecan kernels (Pawnee).

Peak No.	RT (min)	Molecular Ion (*m/z*)	Fragment Ions (*m/z*)	Neutral Loss (amu)	Tentative Structural Assignment	Reference	Molecular Formula	∆m (ppm)
1	0.65	341.0879	179	162	Caffeic acid hexoside	[9]	C_15_H_18_O_9_	−1.76
2	0.84	331.0684	169	162	Gallic acid hexoside	[9]	C_13_H_16_O_10_	−5.74
3	0.94	481.0643	301	180	HHDP-glucose	[9]	C_20_H_18_O_14_	−5.20
4	1.15	169.0146	125	44	Gallic acid	Standard ^a^	C_7_H_6_O_5_	−5.33
5	1.30	137.0229	107	30	*p*-Hydroxybenzoic acid	[9]	C_7_H_6_O_3_	7.30
6	2.05	783.0727	481-301	302-482	Bis-HHDP-glucose (pedunculagin/casuariin isomer) *	[30]	C_34_H_24_O_22_	−5.87
7	2.75	451.1263	289	162	Catechin hexoside	[9]	C_21_H_24_O_11_	−5.10
8	3.13	451.1242	289	162	Catechin hexoside	[9]	C_21_H_24_O_11_	−0.44
9	3.20	783.0684	481-301	302-482	Bis-HHDP-glucose (pedunculagin/casuariin isomer) *	[30]	C_34_H_24_O_22_	−0.38
10	3.27	951.0760	907-783-481-301	44-168-470-650	HHDP-valoneoyl-glucose (praecoxin A/platycariin isomer) *	[30]	C_41_H_28_O_27_	−2.10
11	3.40	577.1337	425	152	Procyanidin dimer B-type [(E)C→B→(E)C]	[9]	C_30_H_26_O_12_	1.56
12	3.75	183.0310	124	59	Methyl gallate ^∆^	[31]	C_8_H_8_O_5_	−8.74
13	3.90	865.2006	577-289	288-576	Procyanidin trimer (C1) [(E)C→B→(E)C→B→(E)C]	[9]	C_45_H_38_O_18_	−3.01
14	3.97	483.0773	331-169	152-314	Digalloyl-glucose	[30,32]	C_20_H_20_O_14_	0.41
15	4.04	289.0732	245-205-179	44-84-110	(+)-Catechin	Standard	C_15_H_14_O_6_	−6.92
16	4.59	633.0756	301	332	Galloyl-HHDP-glucose (strictinin/isostrictinin)	[30,32]	C_27_H_22_O_18_	−4.42
17	5.45	785.0768	483-301	302-484	Digalloyl-HHDP-glucose (tellimagrandin I)	[30,32]	C_34_H_26_O_22_	8.92
18	5.64	289.0722	245-205-179	44-84-110	(−)-Epicatechin	Standard	C_15_H_14_O_6_	−3.46
19	5.78	463.0508	301	162	Ellagic acid hexoside	[9]	C_20_H_16_O_13_	1.08
20	6.36	300.9961	217	84	Ellagic acid	Standard	C_14_H_6_O_8_	7.64
21	6.51	433.0413	301	132	Ellagic acid pentose	[9]	C_19_H_14_O_12_	−1.39
22	6.64	565.2089	403	189	Glansreginin B *	[33]	C_24_H_38_O_15_	7.78
23	6.70	615.1000	463-301	152-314	Digalloyl ellagic acid	[9]	C_28_H_24_O_16_	−2.28
24	6.79	477.0692	315-300	162-177	Methyl ellagic acid hexoside	[9]	C_21_H_18_O_13_	−4.82
25	6.90	609.1041	301	308	Ellagic acid rutinoside #	[34]	C_26_H_26_O_17_	8.37
26	7.02	441.0782	289	152	(Epi)catechin gallate	[9]	C_22_H_18_O_10_	9.07
27	7.21	491.0811	328	163	Dimethyl ellagic acid hexoside	[9]	C_22_H_20_O_13_	3.05
28	7.37	649.0724	605-481-301	44-168-348	Valoneoyl-glucose *	[35]	C_27_H_22_O_19_	−7.24
29	7.44	447.0595	301	146	Ellagic acid rhamnoside #	[36]	C_20_H_16_O_12_	−6.93
30	7.48	315.0153	301	14	Methyl ellagic acid	[19]	C_15_H_8_O_8_	−3.81
31	7.51	447.0578	315	132	Methyl ellagic acid pentose	[9]	C_20_H_16_O_12_	−3.13
32	7.79	592.2066	403-343-241	189-249-351	Glansreginin A *	[33]	C_28_H_35_NO_13_	−6.08
33	7.86	585.0507	433-301	152-284	Ellagic acid galloyl pentose	[9]	C_26_H_18_O_16_	1.71
34	8.09	457.0808	293-163	164-294	Epigallocatechin gallate (EGCG)	Standard	C_22_H_18_O_11_	−8.09
35	8.28	487.0124	469-425-301	18-62-186	Valoneic acid dilactone hydrate	[9]	C_21_H_12_O_14_	5.13
36	8.44	599.0625	447-315	152-284	Methyl ellagic acid galloyl pentose	[9]	C_27_H_20_O_16_	8.01
37	8.67	599.0670	447-315	152-284	Methyl ellagic acid galloyl pentose	[9]	C_27_H_20_O_16_	0.50
38	8.86	599.0684	447-315	152-284	Methyl ellagic acid galloyl pentose	[9]	C_27_H_20_O_16_	−1.84
39	9.17	329.0271	314-301	15-28	Dimethyl ellagic acid	[9]	C_16_H_10_O_8_	7.90
40	9.34	329.0325	314-301	15-28	Dimethyl ellagic acid	[9]	C_16_H_10_O_8_	−8.51

^a^ Standard, this compound was further confirmed by standard material; #, compounds found for the first time in genus *Carya*; *, compounds found for the first time in *Carya illinoinensis*; ^∆^, compounds found for the first time in pecan kernels; E(C), (epi)catechin; B, B-type linkage.

**Table 2:** The correlation between phenolics and antioxidant capacity.

Antioxidant Capacity	TPC	CT	TFC	Individual Phenolics ^1^				
				GA	C	EC	EGCG	EA
DPPH	0.748 **	0.442 **	0.426 **	0.538 **	0.799 **	0.721 **	0.469 **	0.471 **
ABTS	0.841 **	0.497 **	0.506 **	0.662 **	0.871 **	0.851 **	0.597 **	0.606 **

^1^ GA: gallic acid; C: (+)-catechin; EC: (−)-epicatechin; EGCG: epigallocatechin gallate; EA: ellagic acid; **: correlation is significant at the 0.01 level.

**Table 3:** Sample list and description.

Sample Name	DAFB ^1^	Sample Stage	Description ^2^	Daily Mean Temperature of Sampling Interval (°C) ^3^	Daily Mean Sunshine Duration of Sampling Interval (h) ^3^
S1	85	Water stage	Appearance of the kernel	32.09	10.34
S2	95	Water stage	Quick expansion of the kernel	30.38	8.38
S3	105	Water stage	Quick expansion of the kernel	30.73	10.35
S4	115	Milk stage	Kernel became milky	29.67	6.90
S5	125	Milk stage	Kernel became milky	26.69	9.04
S6	135	Dough stage	Kernel became oily	24.30	5.10
S7	145	Dough stage	Kernel became oily	22.80	6.47
S8	155	Dough stage	Kernel ripening	21.93	3.03
S9	165	Kernel stage	Kernel ripening	18.27	2.42
S10	175	Kernel stage	Over ripeness	19.12	1.00

^1^ DAFB: days after full blossoming; ^2^ This description applies to cultivars Stuart and Shaoxing, while cultivars Pawnee, Wichita, and Jinhua are slightly different; ^3^ Daily mean temperatures and sunshine durations of sampling interval were calculated using data of 10 days between the intervals, for example, data of 85 days was calculated using data of 74–85 days, and so on.
